# First determination of some phenolic compounds and antimicrobial activities of Geranium ibericum subsp. jubatum: A plant endemic to Turkey

**DOI:** 10.3906/kim-2005-38

**Published:** 2021-02-17

**Authors:** Mehmet Emin ŞEKER*, Emriye AY, Ayça AKTAŞ KARAÇELİK, Rena HÜSEYİNOĞLU, Derya EFE

**Affiliations:** 1 Espiye Vocational School, Giresun University, Giresun Turkey; 2 Şebinkarahisar School of Applied Science, Giresun University, Giresun Turkey

**Keywords:** *Geranium ibericum* subsp. *jubatum*, phenolic compounds, endemic plant, antimicrobial activity, chromatography

## Abstract

This paper includes the results of the first study about the phenolic characteristics and antimicrobial analyses of *Geranium ibericum* subsp. *jubatum* species found in Turkey *.* In the present work, the phenolic contents of different parts of the *G. ibericum* (flower, root, leaf) were determined with high-performance liquid chromatography (HPLC)–DAD (diode-array detector) and liquid chromatography (LC)–MS/MS (mass spectrometry). The following phenolic compounds were investigated: catechin, protocatechuic acid, gallic acid, ellagic acid, chlorogenic acid, 4-hydroxybenzaldehyde, *p* -coumaric acid, rutin, naringenin, kaempferol *.* Based on the results obtained, the roots and flowers of the plant are found to be very rich in ellagic acid (3473.57 µg g^-1^ dry plant) and catechin (2228.76 µg g^-1^ dry plant). The amount of chlorogenic acid (54.570 µg g^-1^ dry plant) is also high in the roots. The amounts of protocatechuic acid (122.5 µg g^-1^ dry plant) and gallic acid (725.34 µg g^-1^ dry plant) are high in the leaves. In addition, the total extract of *G. ibericum* obtained from leaf, flower, and root was tested against 6 gram-negative bacteria and *Candida albicans* . The *G. ibericum* extract was nearly as effective as commercial antibiotics at some concentrations (500-750 µg µL^-1^) for *Acinetobacter baumannii* , *Klebsiella pneumonia* , *Proteus mirabilis* , and *Bacillus cereus* .

## 1. Introduction

The genus *Geranium* is taxonomically classified in the Geraniaceae family, which contains a total of 840 species. This genus is the largest in the Geraniaceae family. It is represented by approximately 430 annual and perennial plant species which are widely distributed throughout the world, especially in temperate regions and tropical mountains [1–4].

Numerous studies have been conducted on the chemical contents of the subspecies of the genus *Geranium* . According to these studies, phenolic compounds are dominant in *Geranium* , but the most-studied compounds are tannins, flavonoids, and phenolic acids [3,5,6].

Many articles have been published recently on the biological effects of extracts and essential oils of the *Geranium* and their use in medicine, food flavoring, perfumery, and cosmetics [7]. Many biological activities, such as antihelmintic activity and antihepatotoxic activity, were summarized in a review compiled by Graça et al. [8].

According to some studies, many *Geranium* spp. have a wide range of uses. They have been shown to exhibit antioxidant, antibacterial, antiprotozoal, antiviral, antidiabetic, antileishmanial, HIV-RT enzyme inhibitor, antiallergic, antiinflammatory, and antihepatotoxic effects [3,8–10]. In addition, *Geranium* spp. have traditionally been used in antiseptic treatments for wounds, and infusions of the plants have been used as an antipyretic [6]. For example, it has been noted that *G. macrorrhizum* has shown significant hypotensive activity in anesthetized cats [1]. In Japan, *G. thunbergii* has been approved as an official antidiarrheal drug [11,12].

In Turkey, the *Geranium* genus is represented by 39 taxa, 9 of which are endemic. The endemic percentage is 23% [4].

*G. ibericum* , which is endemic to Turkey, has been grown in the northwest and northeast of Turkey, and in the Caucasus and Iran. It is known as “turnagagası” among the local people [13]. Figen et al. mentioned the wound healing properties of *G. ibericum* in their study [14]. However, no published reports of its chemical constituents have been encountered in the literature.

Due to their different therapeutic benefits and traditional uses, research has been performed on the biological and phytochemical aspects of *Geranium* species. Even though simple phenolics, flavonoids, and essential oils have been isolated from *Geranium* species, there have been no reports on the phenolic character and antimicrobial capacity of *G. ibericum.*

This work represents the first analysis of the phenolic constituents of *G. ibericum* extract and determination of its antimicrobial activity.

## 2. Materials and methods

### 2.1. Plant material

*G. ibericum* specimens were collected by Rena Hüseyinoğlu in Eğribel Pass, Giresun, Turkey in June 2018 (40°27′59″N, 38°41′55″E, 2509.53 m a.s.l.). The voucher specimen was deposited in the Herbarium of the Faculty of Science, Ondokuz Mayıs University, Samsun, Turkey. Specimens were identified by Prof. Dr. Erkan Yalçın, Ondokuz Mayıs University, Samsun, Turkey (herbarium number of specimens: 7915).

### 2.2. Chemicals

2,4,6-Tris(2-pyridyl)-s-triazine (TPTZ), FeCl3.6H2O, and analytical grade phenolic standards (catechin, protocatechuic acid, gallic acid, ellagic acid, gentisic acid, chlorogenic acid, *p* -hydroxybenzoic acid, chicoric acid, 4-hydroxybenzaldehyde, *p* -coumaric acid, rosmarinic acid, rutin, oleuropein, naringenin, quercetin, kaempferol, and hesperidin), dimethyl sulfoxide (DMSO), ofloxacin (OFX), netilmicin (NET), sulbactam (SCF), and maxipime were obtained from Sigma-Aldrich (St. Louis, MO, USA). Mueller Hinton Broth (MHB) and Mueller Hinton Agar (MHA) were obtained from Merck (Kenilworth, NJ, USA).

### 2.3. Preparation of extracts from plant parts

The flowers, leaves, and roots of the plant samples were separated, cleaned, and dried in a hot-air oven at 40 °C for 48 h. Methanol was used as the solvent for the Soxhlet extraction process. For Soxhlet extraction, 10 g samples (leaf, flower, and root) of different plant sections were milled in the blender and placed in the Soxhlet cartridge, 250 mL methanol was added to the apparatus, and the system was operated. After this process, the solvent mixture was filtered, and then the solvent was removed by rotary evaporator at 40 °C, 175 mbar. Finally, the samples were dissolved in 25% methanol–water phase for HPLC analysis [15]. Each sample was extracted three times and studied with three parallel samples.

### 2.4. Construction of calibration curves

Absorption results against five concentrations were obtained for each sample. The calibration graph was prepared in the 0.1–5.0 µg mL^-1^ range for low-concentration compounds. Samples were diluted for quantification of high-concentration compounds and quantified according to calibration curves prepared at appropriate intervals.

### 2.5. Validation of method

Reproducibility and accuracy of the method were checked by spiking standards to a solution containing samples at a concentration of 10 mg mL^-1^ of each phenolic compound. The reproducibility and the accuracy of the method were measured from 10 replicate injections. The relative standard deviations (RSDs) for reproducibility are given in Table 1. Relative errors, used to represent accuracy, were between 0.67% and 2.41%.

**Table 1:** Analytical specifications of selected phenolic compounds.

No	Compound	Calibration equationy = ax + b (a)	R2	Linear range(μg mL^-1^)	LODa	LOQa
1	Catechin	y = 4.6834x + 0.6087	0.9980	10.00–200.00	0.0019	0.0057
2	Protocatequic acid	y = 2.0259x + 0.7296	0.9961	5.00–100.00	0.0097	0.0293
3	Ellagic acid	y = 0.3008x + 0.1022	0.9983	10.00–200.00	0.1037	0.3143
4	Gallic acid	y = 1.0597x + 0.6532	0.9977	5.00–100.00	0.0316	0.0957
5	Chlorogenic acid	y = 2.1609x + 0.8897	0.9959	1.50–50.00	0.0080	0.0241
6	4-Hydroxybenzaldehyde	y = 1.0554x + 0.3555	0.9993	1.50–50.00	0.0426	0.1292
7	p-Coumaric acid	y = 0.7531x + 0.5186	0.9989	1.50–50.00	0.0961	0.2913
8	Rutin	y = 4.6524x + 7472	0.9921	1.50–50.00	0.1102	0.3452
9	Naringenin	y = 2.2325x + 0.6321	0.9972	1.50–50.00	0.0245	0.0764
10	Kaempferol	y = 2.2324x + 1.2264	0.9967	1.50–50.00	0.0197	0.0632
11	Taxifolin	y = 8486.7x –255.07	0.9947	1.50–50.00	0.2078	0.6296

Linearity of the HPLC–DAD system was evaluated by using the least square method. The linearity of the method was very good for all standards.

Injection was made 10 times at a detectable concentration for detection and quantification limits (LOD based on 3s and LOQ based on 10s, respectively) and calculated using standard deviations.

Recovery of the extraction procedure was performed by using spiked samples on plant samples. Two known concentrations of phenolic standards were added to the plant samples before extraction. The same extraction procedure was applied to the spiked samples under the same conditions. Recovery of the extraction procedure varied between 76% and 101% at two different concentrations (5 mg L^-1^ and 10 mg L^-1^). The results show that the extraction procedure was quite good. All validation parameters are given in Table 1 and Table 2.

**Table 2:** The amounts of phenolic compounds (µg g–1 dry plant)

Phenolics	Root	Leaf	Flower
Catechin	2228.76	ND	964.20
Protocatechuic acid	24.48	122.51	81.30
Ellagic acid	75.22	445.44	3473.57
Gallic acid	ND	725,34	506.97
Gentisic acid	ND	ND	ND
Chlorogenic acid	54.57	0.40	0.94
p-Hydroxybenzoic acid	ND	ND	ND
Chicoric acid	ND	ND	ND
4-Hydroxybenzaldehyde	0.15	0.55	0.19
p-Coumaric acid	0.82	1.60	2.38
Rosmarinic acid	ND	ND	ND
Rutin	0.08	0.51	0.87
Oleuropein	ND	ND	ND
Naringenin	0.15	ND	0.20
Quarcetin	ND	ND	ND
Kaempferol	0.77	1.03	3.82
Hesperidin	ND	ND	ND
Taxifolin	4.64	ND	ND

### 2.6. Iron (III) reduction/antioxidant force (FRAP) method

The method used in this study is based on the measurement of the absorbance of the TPTZ-Fe (II) complex [16,17]. The activity of all samples was determined as micromolar TEAC (Trolox equivalent antioxidant capacity) by preparing the calibration graphic obtained using Trolox in the range of 31.25–1000 µM. All samples were prepared by diluting 1:100 from the stock solution. 

Briefly, 50 µL of the sample was mixed with 1.5 mL of FRAP reagent (prepared by mixing acetate buffer, TPTZ, and FeCl3.6H2O); the absorbance was read at 595 nm after 20 min incubation at room temperature. The absorbance of all solutions was read against pure water.

### 2.7. HPLC–DAD analysis

The reverse-phase C18 column (150 mm × 4.6 mm, 5μm; Fortis) was used for the chromatographic separation of compounds. A slight modification was made in the mobile phase reported in the literature, which consisted of A: 2% acetic acid–ultrapure water, and B: 50%–50% acetonitrile–ultrapure water including 0.5% acetic acid. The following mobile phase gradient program was used for the separation of phenolic compounds: 0–8 min (7% B), 8–18 min (12% B), 18–23 min (23% B), 23–25 min (40% B), 25–35 min (45% B), 35–40 min (55% B), 40–43 min (92% B), and 43–46 min (25% B) [15].

The injection volume was adjusted to 20 μL, the mobile phase flow rate to 0.7 mL min–1, and the column temperature to 25 °C. The diode-array detector (DAD) was operated at four different wavelengths (260, 280, 308, 324 nm). The wavelengths determined in DAD were selected according to the maximum absorption wavelengths by examining the spectra of phenolic standards.

### 2.8. LC–MS/MS analysis

The ODS Hypersil (4.6 × 250 mm, 5 µm) column was used in liquid chromatography (LC)–MS/MS (mass spectrometry) analyses. For mobile phases, water containing 0.1% formic acid, and B methanol was used. The mobile phase solvent program was adjusted to 0 and 0–1 min 100% A, 1–22 min 5% A, 22–25 min 5% A, and 25–30 min 100% B. The flow rate was set to 0.7 mL min–1, the column temperature to 30 °C, and the injection volume was 20 µL.

### 2.9. Determination of antimicrobial activity of the extracts

First, the extracts of *G. ibericum* obtained from leaf, flower, and root were tested against 6 Gram-negative bacteria ( *Acinetobacter baumannii* [ATCC BAA-747], *Klebsiella pneumonia* [ATCC 13883], *Citrobacter freundii* [ATCC 43864], *Enterobacter aeruginosa* [ATCC 3048], *Pseudomonas aeruginosa* [ATCC27853], and *Proteus mirabilis* [ATCC 43071]) and 6 Gram-positive bacteria ( *Streptococcus pneumonia* [ATCC 49619], *Staphylococcus aureus* [ATCC 25923], *Bacillus cereus* [ATCC 10876], MRSA ATCC 67101, *Bacillus subtilis* [ATCC 6633], *Staphylococcus epidermidis* , and *Candida albicans* [ATCC 10231]). They were supplied by Erzurum Technical University, Molecular Biology and Genetics Laboratory. The microorganisms were cultured aerobically in Mueller Hinton Broth (MHA, Merck 1.10293) and on Mueller Hinton Agar (MHA, Merck 1.05437) at 37 °C. The total extracts were studied at the second and third tests as there had been no differences between root, leaf, and flower extracts in terms of antimicrobial activity. The antimicrobial activity and minimal inhibitory concentrations (MIC) were determined by the disc diffusion and minimum dilution methods, respectively. The experiments were performed according to the method described by [18]. The extract was diluted to 300–1000 µg µL^-1^ with DMSO (20%). Ofloxacin (OFX) (10 μg disc–1), netilmicin (NET30) (30 μg disc–1), and sulbactam (SCF) (30 kg disc–1) were used as positive references for the disc diffusion method. Maxipime (Bristol-Myers Squibb, New York, NY, USA) in concentrations of 7.81–500 µg µL^-1^ was used as a positive reference for minimal inhibitory concentration determination; 10 μL of DMSO (20%) was the negative reference. Each experiment was performed three times. 

## 3. Results and discussion

The total phenolic content analysis and antioxidant capacity (using ABTS and DPPH methods) of *G. ibericum* were determined spectrophotometrically as reported by Ay et al. [19]. As in that study, the FRAP method used to determine the antioxidant capacity was applied in paper and the results were evaluated. In addition, in the continuation of these studies, we aimed to determine the phenolic characteristics of these endemic species extracts with chromatographic studies and antimicrobial activities. Since it dissolves low-molecular–weight polyphenols, methanol was used as the extraction solvent. It should be noted that the compounds detected by the chromatographic method and the antimicrobial capacity assessment belong only to the methanol extract, taking into account that the components to be dissolved will change as the solvent changes [20].

### 3.1. Iron (III) reduction/antioxidant force (FRAP) method results

**Figure 1:**
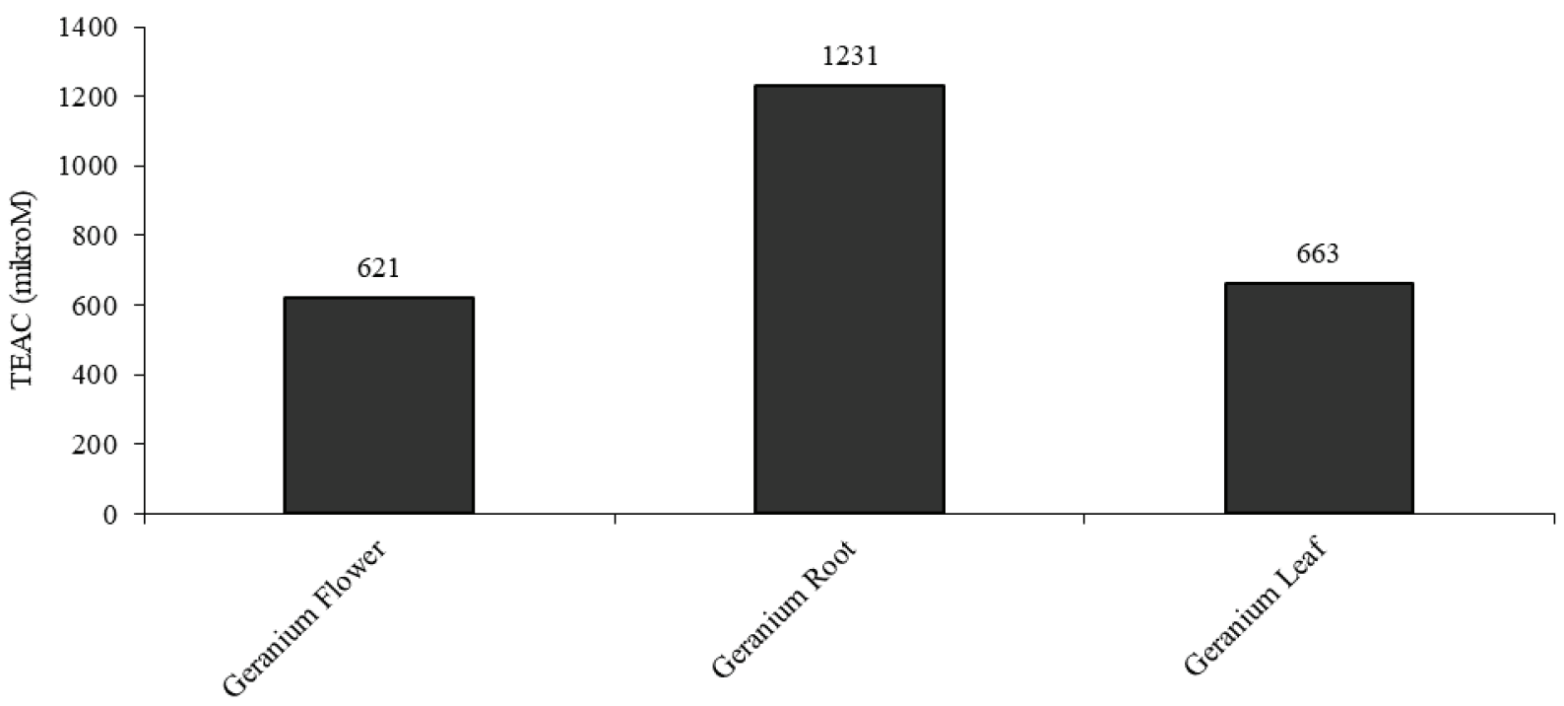
Fe (III) reduction / antioxidant strength (FRAP) values of the Geraniumibericum flower, root and leaf extracts (μM TEAC; Trolox equivalent antioxidant capacity).

A calibration curve based on absorbance measurements was constructed by using different concentrations of Trolox. Using this curve, antioxidant activity was expressed as Trolox equivalent antioxidant capacity (TEAC, μM); the graph of the column is given in Figure 1. All samples used in the FRAP method were analyzed by diluting 1:100 from the stock solution.

The iron (III) reduction forces of the samples are 207, 410, and 221 µM TEAC for flower, root, and leaf, respectively and are shown in Figure 1. When the iron (III) reduction/antioxidant strengths of the extracts were compared, the root extract had the highest iron extracting potential and the highest antioxidant activity. The lowest iron (III) reduction/antioxidant strength was found in the flower extract. These results are similar to those of the previous study involving ABTS and DPPH methods [19].

### 3.2. Determination of phenolic compounds by chromatographic methods

**Figure 2:**
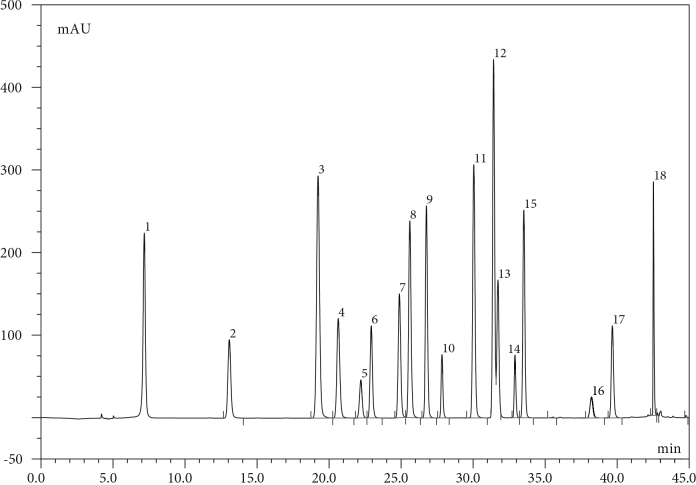
HPLC-DAD chromatogram of 50 ppm mix standards. 1: Galllic Acid, 2: Protocatequic Acid, 3:Protocatequic Aldehyde, 4:p-hydrox Benzoic Acid, 5: Catechin, 6: Chlorogenic Acid, 7: Vanillic Acid, 8: 4-Hydroxybenzaldehyde , 9: Syringic Acid, 10: Epicetechin, 11: Vanillin, 12: p-Coumaric Acid, 13: Ellagic Acid, 14: Rutin, 15: Taxifolin, 16: Benzoic Acid,17: Naringenin, 18: Kaempferol.

The phenolic contents of *Geranium* are given in Table 3. Chromatograms of the standard mixture and extracts are given in Figures 2 and 3a,b,c.

**Table 3:** Recovery and RSD % value of study.

Compounds	RSD(%)Root	Recovery(%)Root	RSD(%)Leaf	Recovery(%)Leaf	RSD(%)Flower	Recovery(%)Flower
Catechin	0.98	101	1.22	98	0,99	97
Gallic acid	1.07	99	1.85	97	1.42	97
Ellagic acid	1.58	96	1.63	98	1.51	95
Protocatechuic acid	2.18	98	2.42	96	2.27	96
Chlorogenic acid	2.24	92	2.15	88	2.63	94
4-Hyroxybenzaldehyde	2.47	83	3.05	76	2.19	82
p-Coumaric acid	2.82	94	2.61	92	2.37	89
Rutin	1.91	76	1.95	78	1.83	82
Naringenin	2.14	90	2.40	87	2.38	91
Kaempferol	2.14	86	1.92	89	1.79	83
Taxifolin	0.81	97	1.28	96	1.17	95

**Figure 3a:**
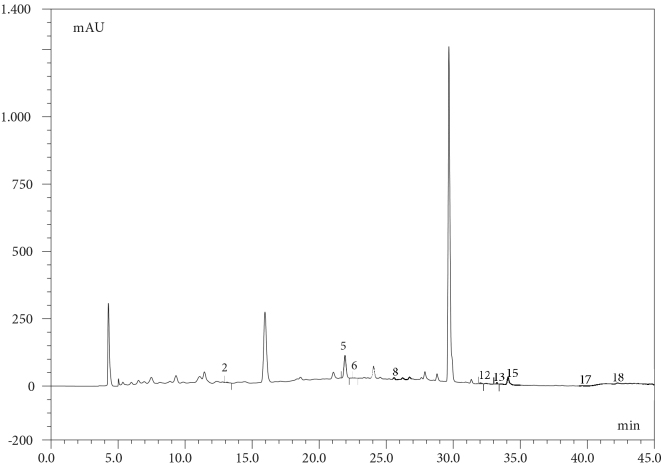
HPLC-DAD Chromatogram of Geranium ibericum subsp. jubatum Root. 2: Protocatequic acid, 5: Catechin, 6: Vanillic acid, 8: 4-Hydroxybenzaldehyde, 12: p-Coumaric acid, 13: Ellagic acid, 15: Taxifolin, 17: Naringenin, 18: Kaempferol.

**Figure 3b:**
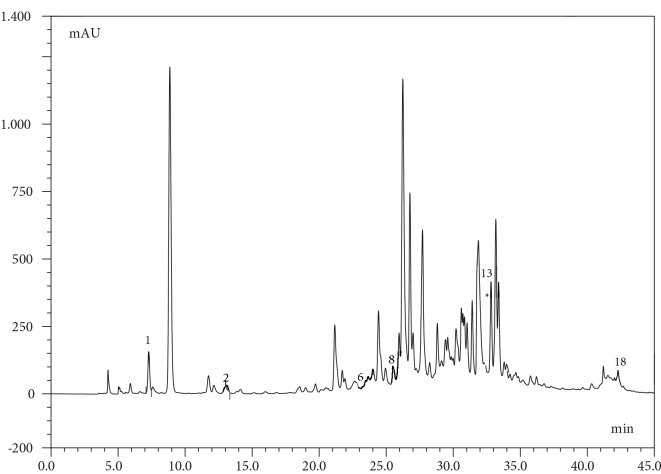
HPLC-DAD Chromatogram of Geranium ibericum subsp. jubatum Leaf. 1: Gallic acid, 2: Protocatequic acid, 6: Vanillic acid, 8: 4-Hydroxybenzaldehyde, 13: Ellagic acid, 18: Kaempferol. *Since the amounts of routine and p-Coumaric acid are low and their peaks coincide with other peaks in the chromatogram, their quantities were determined by LC-MS / MS.

**Figure 3c:**
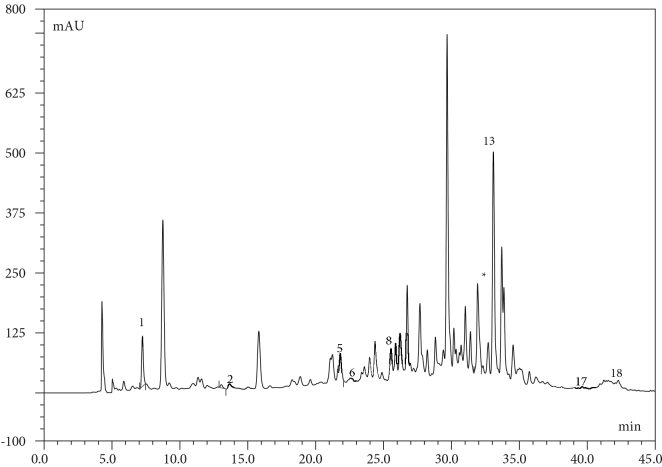
HPLC-DAD Chromatogram of Geranium ibericum subsp. jubatum Flower. 1: Gallic acid, 2: Protocatequic acid, 5: Catechin, 6: Vanillic acid, 8: 4-Hydroxybenzaldehyde, 13: Ellagic acid, 17: Naringenin, 18: Kaempferol, Rutin and p-coumaric acid. *Since the amounts of routine and p-coumaric acid are low, and their peaks coincide with other peaks in the chromatogram; they are quantified by LC–MS/MS.

The results show that the amounts of ellagic acid, catechin, gallic acid, protocatechuic acid, and chlorogenic acid are remarkably high. The roots and flowers of the plant are very rich in ellagic acid and catechin. The amount of chlorogenic acid in the root is high, and the amounts of protocatechuic acid and gallic acid in the leaves are high. There is also a high amount of gallic acid in the flowers. *p* -Coumaric acid and kaempferol amounts were higher than those of other phenolics. According to the results of the phenolic contents, the antioxidant strength of the root is significantly higher than that of the leaves and flowers.  According to the results given in Table 2, the highest phenolic content can be seen in the root and flower. 

A simple extraction was carried out with the samples, and the quantity of many phenolic compounds was determined by HPLC–DAD and LC–MS/MS. This study was the first to investigate the phenolic content of an endemic *Geranium* species. Although the essential oil content for similar *Geranium* species has been examined in the literature, the phenolic component content has not frequently been studied. In one of the studies, 17 phenolic compounds were searched for, and 8 of them could be determined. The amounts of phenolic compounds found in the sample study in mg g^-1^ as follows: caftaric acid 1.30, caffeic acid 2.41, hyperoside 1.64, isoquercitrin 2.58, rutin 1.71, quercitrin 0.42, quercetin 0.82, kaempferol 0.19 [21]. In another study with *G. carolinianum* , 5 bioactive components were investigated by performing water extraction; only gallic acid and elagic acid were analyzed. The gallic acid results found in that study are about 10 times higher than what we found. The elagic acid results were approximately the same [22].

In a similar study, some of the main phenolics of the *G. sibiricum* plant were determined. In this study, the amount of gallic acid was higher than the amount we detected [23]. Apart from this, there was no commonality in the determined compounds. In our study, 18 phenolic compounds were searched for, 11 of which could be quantified. The components with the highest amounts were ellagic acid, catechin, gallic acid, and protocatechuic acid. Total ellagic acid, catechin, gallic acid, and protocatechuic acid amounts were 3.99, 3.19, 1.23, and 0.23 mg g^-1^, respectively, in root + leaf + flower.

Ellagic acid has been reported to play a protective role against the negative effects of various stress sources. It has also been shown to activate apoptotic pathways, leading to the reduction of cancer and other chronic diseases [24].

Catechin, an important flavonoid, has been demonstrated to effectively inhibit metastases, which may result from inhibition of metalloproteinases [25]. It has also been shown that catechin inhibits lipid oxidation by delaying the consumption of lipid-soluble antioxidants and is an effective antioxidant in human blood plasma [26].

Gallic acid, a polyphenol, has been shown to inhibit carcinogenesis in animal experiments and in vitro cancer cell lines. In addition to drug development, it reduces the risk of developing cancer when taken as a food supplement [27].

When studies on *Geranium* spp. are examined, it is seen that some subspecies belonging to the *Geraniaceae* family contain a substance called geraniin that has many important biological activities such as anticancer, antimicrobial, antiviral, and antihyperglycemic activities [28]. When geraniin is hydrolyzed, it produces gallic acid, ellagic acid, corilagin, and brevifolin carboxylic acid [29]. It has been found that these metabolites released by the hydrolysis of geraniin have a range of bioactive properties including antioxidant and free radical scavenging activity [30,31]. However, studies have not shown that all *Geranium* spp. contain these four components as the main components [1].

### 3.3. Antimicrobial activity

The antimicrobial effects and MIC values of the *G. ibericum* subsp. *ju* *batum* extract (GIJE) were determined by the disc diffusion method and minimum dilution methods at concentrations ranging from 300 to 1000 µg mL^-1^. (Table 4). Since no differences have been observed between different parts of the plant, it was decided to study the total *G. ibericum* extract (GE). Contrary to expectations, the highest dose (1000 µg mL^-1^) showed no antimicrobial activity and the second highest dose showed effectiveness against only 2 bacteria ( *Acinetobacter baumannii* and *Proteus mirabilis* ). GIJE is thought to be completely insoluble and precipitated at high concentration, and consequently showed no antimicrobial activity. The MIC values were as follows: 300 µg mL^-1^ for *P. mirabilis, B. cereus* , and *S. epidermidis* ; 400 µg mL^-1^ for *A. baumannii* , *K. pneumonia* , *E. aeruginosa* , *P. aeruginosa* , *S. aureus* , *B. subtilis* , and *C. albicans* ; 500 µg mL^-1^ for *C. freundii* , *S. pneumonia* , and MRSA ATCC 67101. Significant differences were not observed in terms of being gram-negative or gram-positive. GE was nearly as effective as commercial antibiotics in some concentrations against *A. baumannii* , *K. pneumonia* , *P. mirabilis* , and *B. cereus.* Lis-Balchin and Deans [32] reported that the extracts obtained from 8 different members of the Geraniaceae family showed significant antibacterial activity against at least 18 out of 25 bacteria and very slight activity against fungi. Radulović et al. [33] studied the volatile oils obtained from *G. sanguineum and G. robertianum* . The oils showed strong action against *E. coli* and *A. fumigatus* . Giongo et al. [34] showed that the antimycobacterial and antimicrobial activities of geranium-oil–loaded nano-capsules were similar to those of free oil. The geranium oil-loaded nano-capsules were not effective in inhibiting the formation of germ tubes of *C. albicans* [32]. However, GE showed antimicrobial activity against *C. albicans* in this study. In the literature, there are a few studies about the antimicrobial activity of the *Geranium* species, but none of them concern *G. ibericum* . This study is the first study which evaluates the antimicrobial activity of GIJE.

**Table 4:** Antimicrobial activity of the Geranium ibericum subsp. jubatum.

Bacteria	Concentrations (µg µ^-1^)						MIC	Negativecontrol	Standard antibiotic discs		
	1000	750	600	500	400	300			OFX	NET30	SCF
Acinetobacter baumanni	-	18	12	10	8	-	400	-	18	10	10
Klebsiella pneumoniae	-		17	14	9	-	400	-	19	11	16
Citrobacter freundii	-	-	10	8	-	-	500	-	24	24	20
Enterobacter aeuriginosa	-	-	8	8	7	-	400	-	19	21	18
Pseudomonas aeruginosa	-	-	10	8	7	-	400	-	21	20	18
Proteus mirabilis	-	18	14	13	10	7	300	-	20	17	15
Streptococcus pneumoniae	-	-	11	8	-	-	500	-	21	21	12
Staphylococcus aureus Bacillus cereus	--	--	1017	914	710	-8	400300	--	2123	2115	1616
MRSA ATCC 67101	-	-	12	8	-	-	500	-	23	21	14
Bacillus subtilis Staphylococcus epidermidis	--	--	1514	1012	810	-7	400300	--	2021	2021	2115
Candida albicans	-	-	10	9	8	-	400	-	20	19	17

*OFX: Ofloxacin (10 μg disc^-1^), NET30: netilmicin (30 µg disc–1), SCF: (30 µg disc^-1^) **Negative control: DMSO (%20) ***MIC (minimal inhibition concentration) was calculated as µg mL^-1^

## 4. Conclusion

In this study, the phenolic characters and antimicrobial activity of extracts of the different parts of the endemic *G. ibericum* were explored. Eighteen phenolic compounds were investigated; 11 of them were detected with chromatographic methods. The antioxidant and antimicrobial activities of the plant were determined. *Geranium* species are used in alternative medicine because of these properties. The main components that are generally isolated in this plant are catechin, ellagic acid, gallic acid, and protocatechuic acid. These components are known to have important functions. According to the results of antimicrobial activity experiments, the use of geranium extract seems to be a promising tool in overcoming pathogen microorganisms.

Further studies of other phenolic compounds (such as geraniin) and essential oils of this species should be carried out; results obtained from such a study in Turkey would make a comparison possible with other *Geranium* species in the world.

## Author’s contributions

**Şeker, M.E.:** Analyses and evaluation of chromatographic studies, organization of the study. 

**Ay, E.:** Collection and extraction of the *Geranium* species; organization of the study. 

**Karaçelik, A.A.:** Analyses and evaluation of chromatographic studies, determination of antioxidant activity. 

**Hüseyinoğlu, R.:** Identification and collection of the *Geranium* species. 

**Efe, D.:** Analysis and evaluation of antimicrobial activity. All of the authors approved the final manuscript.
